# Supplementary material for: Understanding the relationship between safety beliefs and knowledge for cognitive enhancers in UK university students
Source: PLoS One. 2021 Jan 28;16(1):e0244865. doi: 10.1371/journal.pone.0244865 (PMC7842904; doi:10.1371/journal.pone.0244865)
Supplement: S1 File — (DOCX) [file pone.0244865.s001.docx]

## S1 File. Survey questions.

### Sample characterization questions

Please indicate your gender:

- Male
- Female

Please indicate your age in years and months at the time of this study, for example, 20 years and 4 months.

________________________________________________________________

Please indicate the type of qualification you are currently working towards:

- Undergraduate degree (e.g. BSc)
- Taught postgraduate degree (e.g. MSc)
- Research postgraduate degree (e.g. PhD)

Since beginning your current qualification have you ever taken any of the following drugs with the intent to improve your study results?

Methylphenidate

Amphetamine

Modafinil

Beta-blockers

Rivastigmine

- Yes
- No

*Display This Question: If Since beginning your current qualification have you ever taken any of the following drugs with th... = Yes*

Which of the following have you taken since the start of your current qualification? (tick all that apply)

- Methylphenidate
- Amphetamine
- Modafinil
- Beta-blockers
- Rivastigmine

*Display This Question: If Since beginning your current qualification have you ever taken any of the following drugs with th... = Yes*

Consider all of the drugs selected previously, how frequently have you or do you take them?

- Less than once a year
- Once a year
- Once per term
- Once per month
- Once per week
- More than once per week

### Safety belief questions

Please indicate your agreement with the following statements.

|  | Strongly agree | Agree | Somewhat agree | Neither agree nor disagree | Somewhat disagree | Disagree | Strongly disagree |
| --- | --- | --- | --- | --- | --- | --- | --- |
| It is important to know whether smart drugs are safe to use. |  |  |  |  |  |  |  |
| I know enough about smart drugs to judge whether they are safe to use. |  |  |  |  |  |  |  |
| I think smart drugs are safe to use. |  |  |  |  |  |  |  |

### Sources of information questions

When considering the safety of smart drugs, which of the following sources do you use? (tick all that apply)

- Personal experience
- Experiences of peers or friends
- Information on websites
- Social media
- NICE Guidelines
- Scientific research
- Other (please specify)

For each of the sources, irrespective of whether you use them to make judgments, rate how reliable you believe it is as a source of information on smart drugs.

|  | Extremely unreliable | Moderately unreliable | Slightly unreliable | Neither reliable nor unreliable | Slightly reliable | Moderately reliable | Extremely reliable |
| --- | --- | --- | --- | --- | --- | --- | --- |
| Personal experience |  |  |  |  |  |  |  |
| Experiences of peers or friends |  |  |  |  |  |  |  |
| Information on websites |  |  |  |  |  |  |  |
| Social media |  |  |  |  |  |  |  |
| NICE Guidelines |  |  |  |  |  |  |  |
| Scientific research |  |  |  |  |  |  |  |
| Other (as specified above) |  |  |  |  |  |  |  |

### Knowledge of drug effects

The following section is about modafinil, one of the most commonly used smart drugs.
 
You should not take modafinil if you have the following medical conditions. Indicate whether you believe this to be true or false for the conditions listed.

|  | True | False |
| --- | --- | --- |
| Diabetes |  |  |
| High blood pressure |  |  |
| Irregular heartbeat |  |  |
| Low blood pressure |  |  |
| Migraines |  |  |

Careful monitoring is required if you take modafinil and have the following medical conditions. Indicate whether you believe this to be true or false for the conditions listed.

|  | True | False |
| --- | --- | --- |
| Anxiety |  |  |
| Bipolar disorder |  |  |
| Diabetes |  |  |
| Depression |  |  |
| High blood pressure |  |  |
| Heart problems |  |  |
| Kidney problems |  |  |
| Liver problems |  |  |
| Irritable bowel syndrome |  |  |

This question refers to potential side effects of modafinil. For each of the side effects listed, indicate the frequency with which you think it is likely to occur in individuals taking the drug.

|  | 1 in 10 | 1 in 100 | 1 in 1000 |
| --- | --- | --- | --- |
| Headache |  |  |  |
| Diarrhoea |  |  |  |
| Hayfever |  |  |  |
| Shortness of breath |  |  |  |
| Increased heartbeat |  |  |  |
| Sleepiness |  |  |  |
| Loss of sex drive |  |  |  |
| Difficulty swallowing |  |  |  |
| Vomiting |  |  |  |
| Chest pain |  |  |  |
| Back pain |  |  |  |
| Pins and needles |  |  |  |
| Irritability |  |  |  |
| Abnormal urine or more frequent urination |  |  |  |
| Restlessness |  |  |  |
