# Supplementary material for: Understanding the relationship between safety beliefs and knowledge for cognitive enhancers in UK university students
Source: PLoS One. 2021 Jan 28;16(1):e0244865. doi: 10.1371/journal.pone.0244865 (PMC7842904; doi:10.1371/journal.pone.0244865)
Supplement: S2 File — (DOCX) [file pone.0244865.s002.docx]

**S2 File.** **Understanding the relationship between safety beliefs and knowledge for cognitive enhancers in UK university students.**

**Power analyses**

We used G*Power 3.1.9.6 for macOS (Faul et al., 2007) to estimate the statistical power for our tests of mean differences between groups (users vs. non-users).

*Aim 1 – Number of information sources drawn on.* Using the observed standard deviations (SDs) to estimate the standardised effect size (*d*) for these calculations, and the observed ratio of users to non-users (1:3.9) we determined that our sample size (N = 147) exceeded the total sample size of 118 required for 0.95 power to detect a mean difference of one additional data source in the population using a two-tailed test. To allow for the possibility that observed SDs underestimate the SD in the population, which would result in an over-estimation of statistical power, we re-ran the power calculation using the upper limit for the 95% CI for the SD obtained using 1000 bootstrapped samples (Bias-corrected and accelerated). This revised calculation determined that a total sample of 144 was required (n_1_ = 29, n_2_ = 115) for 95% power. Thus, even with a conservative correction to the assumptions of the power calculation, this suggests that we had excellent power to detect a group difference in the population if, on average, one group used one more source of data than the other.

*Aim 2 – Accuracy of safety knowledge.* For the three knowledge scores (not safe, monitor, side effects) we estimated the sample sizes required to have statistical power of 0.8 to detect an absolute mean difference of 10% of the scale-range. To contextualise the meaning of such an effect, for objective knowledge assessed in a university setting, students differing by this amount would obtain different letter grades. As per Aim 1, we used the observed SDs to estimate the effect size (d) and to specify the ratio of users to non-users (1:3.933). For two of these three mean differences, our sample size (N = 148) exceeded that required for 80% power to detect the specified size of effect, with the total sample sizes required being N = 204 (not safe), N = 106 (monitor) and N = 132 (side effects). Using the same conservative correction to the estimate of the population SD as we used for Aim 1, the samples sizes required for 80% power are 242, 140 and 162, respectively. This suggests that, while the power to detect differences in knowledge about when modafinil should not be taken was somewhat less that we would have hoped for, overall, we had good statistical power to detect a 10% absolute difference in knowledge score in the population.

**Robustness checks for tests of mean difference**

For between-subjects tests of means, homogeneity of variance across groups is a standard assumption of the inferential tests that we used (independent samples t-tests, ANOVA). The effects of violating this assumption are exacerbated when groups have unequal sample sizes. *A priori,* we had no reason to discard the assumption of equal variances and *post hoc* there was little evidence in the sample data of heterogeneity in variances of a degree that would cause concern for our tests (Howell, 2017). Nonetheless, because our group sample sizes were highly uneven, we re-ran our analyses without the assumption of equal variances. This provides a robustness check on the analyses reported in our main manuscript. Table S1 shows that there is little difference between the two types of analyses: in no case does the test-statistic or *p*-value for a given variable differ substantially between these analyses; and running the analyses without the assumption of equal variances does not change the conclusions.

**Table S1.** Comparison of group differences (users vs. non-users) with and without the assumption of homogeneity of variance in the populations of scores.

| Variable tested for mean differences | Equal variances assumed | | |  | Equal variances not assumed | | |
| --- | --- | --- | --- | --- | --- | --- | --- |
|  | t | df | p |  | t | df | p |
| Number of information sources | 1.90 | 145 | .058 |  | 1.99 | 47.36 | .053 |
| Source reliability^a^ | 0.39 | 146 | .695 |  | 0.43 | 50.50 | .669 |
| Knowledge (not safe) | 1.25 | 146 | .179 |  | 0.72 | 44.16 | .190 |
| Knowledge (monitor) | 0.72 | 146 | .471 |  | 0.77 | 48.92 | .445 |
| Knowledge (side effects) | 1.38 | 146 | .171 |  | 1.42 | 46.64 | .163 |

^a^ This between-subjects main effect of use status was examined via the factorial ANOVA reported in the manuscript (Aim 2).

**References**

Faul, F., Erdfelder, E., Lang, A.-G., & Buchner, A. (2007). G*Power 3: A flexible statistical power analysis program for the social, behavioral, and biomedical sciences. *Behavior Research Methods*, *39*, 175-191.

Howell, D.C. (2017). *Fundamental Statistics for the Behavioral Sciences* (9th edition). Cengage Learning**.**
