# Supplementary material for: Understanding the relationship between safety beliefs and knowledge for cognitive enhancers in UK university students
Source: PLoS One. 2021 Jan 28;16(1):e0244865. doi: 10.1371/journal.pone.0244865 (PMC7842904; doi:10.1371/journal.pone.0244865)
Supplement: Attachment — Submitted filename: Response to Reviewers.docx [file pone.0244865.s003.docx]

Response to Reviewers

Reviewer #1: This study examined how beliefs about the safety of cognitive enhancers (CEs), knowledge about their safety, and sources of relevant knowledge differed between users and non-users of CEs. One hundred forty-eight university students completed an online questionnaire. The results showed that 21% of the students had used CEs, and they highly evaluated the safety of CEs compared to non-users. On the other hand, sources of information on CE safety did not differ for users and non-users, suggesting that the higher safety levels perceived by users was not because information sources were different from those of non-users. There was also no significant difference between the groups regarding knowledge of CE safety. In addition, there was no significant correlation between safety beliefs and safety knowledge among CE users. This study addresses an interesting issue, and the finding that the basis for the safety beliefs of CE users is weak has considerable social significance. However, there are concerns, mainly about the validity of the results. The authors should consider the following three issues and make the necessary corrections:

1) Since the findings of this study are based on null results, the interpretation of the results should be performed carefully. In this study, the sample of CE users was only 30 of 148 students who cooperated by completing the online survey. What is the statistical power in this case? One of the important findings of this study is that the number of sources and the degree of utilization of each source regarding CE safety knowledge did not differ between CE users and non-users. However, in some analyses, the p-value was shown to be close to a significant value. Therefore, there is the possibility that the p-value did not become significant because of insufficient statistical power. Thus, the evidence is too weak to conclude that there was no difference in the sources of safety knowledge for CE users and non-users. The result that there was no difference in safety knowledge between the groups should be also considered carefully. The authors should show the results of the power analysis to indicate that the size of the sample was sufficient for detecting a significant difference. Otherwise, data should be collected from more participants (especially CE users) based on sample size determination. The author stated in line 377 that “the overall sample size was sufficient for statistical analysis,” but the basis for this reasoning is unclear.

Thank you for this comment, and for this reminder to report and discuss the precision of our estimation in the manuscript. In keeping with current recommendations for this, we now include confidence intervals for each of the mean difference between users and non-users that we report (Cumming, 2014). This means that readers can see what the likely limits are for the effects that we report, and can judge whether any effects that significance tests may have failed to detect might be sufficiently large to warrant further investigation. You will see from the confidence intervals that we report that our sample size of 148 allowed for reasonable precision in estimation, such that it is highly unlikely that we have failed to detect large effects. Additionally, we now acknowledge and discuss that we have limited power to detect small effects. In keeping with the reviewer’s comment above, we do that in relation to the non-significant effect for which the p-value came closest to .05. We also discuss statistical power, more generally, in our limitations section and include recommendations for other researchers.

2) Although the authors aimed to compare CE users and non-users, there is no table or graph showing the data for each. Although the Results section explains whether the differences were significant, it is important to show which group scored higher. The authors should present data by group.

We have now replaced the original Figure 1 with a figure that divides the data by group. This provides by group data for Aim 1. We have also added an additional Figure (Figure 2) to provide by group data relating to Aim 2. Aim 3 data is provided overall in a table and correlations by group are in the text. Therefore, by group data is now presented for all aims.

3) Human behavior is determined by considering not only risks but also benefits. However, in this study, there were three questions focused only on the risks associated with modafinil; they were included to examine participants’ knowledge regarding the safety of CEs. This approach was also used to examine beliefs about CEs. The authors should explain why they focused only on risks and not benefits.

We have now included a paragraph within the introduction explaining our rationale for this approach. To briefly summarise this here we have explained that when studying drug safety, including safety of CE, the focus is typically on risk rather than benefits, and that the benefits are not very well understood, even in key populations. Additionally, theoretical models, supported by substantial experimental data, indicate that risks or potential losses are more important than benefits or gains, and health behaviour interventions that modulate risk perception, rather than benefits, are more likely to be effective. However, we have also included in the discussion that this restriction to risks is a limitation of the current study.

Additional Requirements

1. Please ensure that your manuscript meets PLOS ONE's style requirements, including those for file naming. The PLOS ONE style templates can be found at:

https://journals.plos.org/plosone/s/file?id=wjVg/PLOSOne_formatting_sample_main_body.pdf

https://journals.plos.org/plosone/s/file?id=ba62/PLOSOne_formatting_sample_title_authors_affiliations.pdf

We have made amendments to the manuscript to bring this in line with the requirements.

2. Please include additional information regarding the survey or questionnaire used in the study and ensure that you have provided sufficient details that others could replicate the analyses. For instance, if you developed a questionnaire as part of this study and it is not under a copyright more restrictive than CC-BY, please include a copy, in both the original language and English, as Supporting Information.

We have now provided a full copy of the survey used as supporting information and made this clear from the text.

3. We note that you have stated that you will provide repository information for your data at acceptance. Should your manuscript be accepted for publication, we will hold it until you provide the relevant accession numbers or DOIs necessary to access your data. If you wish to make changes to your Data Availability statement, please describe these changes in your cover letter and we will update your Data Availability statement to reflect the information you provide.

This is still the case. Our institutional repository will only include published data. Therefore, on acceptance we will publish the data with them and provide the DOI.

Reference

Cumming, G. (2014). The new statistics: Why and how. *Psychological Science*, *25*(1), 7–29. <https://doi.org/10.1177/0956797613504966>
