# Supplementary material for: Understanding the relationship between safety beliefs and knowledge for cognitive enhancers in UK university students
Source: PLoS One. 2021 Jan 28;16(1):e0244865. doi: 10.1371/journal.pone.0244865 (PMC7842904; doi:10.1371/journal.pone.0244865)
Supplement: Attachment — Submitted filename: Response to Reviewers Round 2.docx [file pone.0244865.s004.docx]

**Response to Reviewers**

**Reviewer #1:** This study investigated the relationship between the safety beliefs on the use of cognitive enhancers (CE), the sources of knowledge relating to their safety, and the existence of actual knowledge among its users as well as non-users. Of the three points to which I had drawn attention to, in the first review; the second and third were appropriately addressed, but the first was answered only partially, leading to an inadequacy in this paper. In the first review, I had recommended to the authors to either show that the results of the power analysis based on the present sample size were sufficient for detecting a significant difference or to collect additional data, particularly on CE users, based on sample size determination. However, they neither implemented my recommendations nor explained their rationale for not doing so; but instead showed a 95% confidence interval (CI) for the mean differences. While I agree that it is beneficial to report the 95% CI, this revision is not a solution to the issue that I pointed out, since CI is an index of the accuracy of the differences of mean values rather than an index of statistical power.
One of the main objectives of this study was to clarify whether there were differences in the sources of knowledge on the safety of CE between its users and non-users. However, the results showed no significant differences between the two groups relating to the number of reported sources. Regarding this result, the authors argued that even if a difference actually existed in the number of sources between the CE users and non-users, it could have been just one or two, based on the 95% CI of the mean difference (lines 328–333), which they did not consider as a large difference. However, this interpretation is arbitrary because there is no basis for their considering the difference as not being large.

More importantly, the revised version of Figure 1 shows that compared to CE non-users, its users were more likely to use the experiences of peers and websites as sources of information on CE safety. Also, the opposite trends can be seen for social media, NICE guidelines, and scientific research. The p-value of these differences was around 0.15 in several cases, which suggests some meaningful differences, although it is a small effect. While this is an important trend relating to the objective of this study; the reliability of this difference is unclear because its statistical power was weak. Since the authors were unable to provide a clear answer relating to the study’s main objective, I feel that, as of now, this study is not publication-ready. To overcome the above-mentioned ambiguity, the authors should carry out the research and resubmit it, after increasing the number of participants.

**Response:** We are pleased that the reviewer noted we had addressed two of their concerns with this revision. For the concern about power we have made some further amendments to the manuscript, but we respectively disagree with the reviewer about this issue and explain this fully below.

In keeping with current recommendations for best practice (e.g., Cumming, 2014), we included confidence intervals for effects wherever it was straightforward to do so. There is a direct link between confidence intervals and power calculations because both provide information about possible effects in the population, and both rely on the standard error: the width of a confidence interval is determined by the standard error, as is the power to detect a population effect of a given size. Therefore, when – for example – an analysis has high power to detect a small effect, the confidence interval for that effect will be very narrow; while when the power to detect a moderate or large effect is poor the confidence interval will be very wide. To illustrate, suppose we have a confidence interval for a mean difference of width 9.99 units, and the true effect is 10.00 units. By definition of the CI, we expect 95/100 studies to have confidence intervals that include the true effect of 10.00. it follows from the interval width that those 95/100 studies will all return significant results because the confidence interval must exclude zero (an interval that is 9.99 wide cannot include both 0 and 10.00). Therefore, the probability of obtaining a significant effect exceeds 0.95, and therefore it follows from the definition of statistical power that the power to detect this effect exceeds .95., We regret not making that link explicit in our previous version, but have done so in our current revision, as follows (pages 13 and 14, red font represents the main revision to this portion):

While the mean number of sources used was higher for users (M = 3.43, SD = 1.14) than non-users (M = 2.97, SD = 1.21), this difference was not statistically significant (t(145) = 1.91, *p*=0.058, 95% CI -0.95, 0.01) although the effect was small-to-medium in size (*d*=0.40). Thus, we did not detect a difference between users and non-users in the mean number of information sources used. An implication of the width of the 95% CI for this difference (width just below 1) is that our study had excellent statistical power (>.95) to detect a difference of 1 additional data source.

For none of these measures did the mean number correct differ significantly between users and non-users (Fig 2): Not Safe, *t*(146)=1.35, *p*=0.179, 95% CI –0.69, 0.13; Monitor, *t*(146)=0.72, *p*=0.471, 95% CI -0.72, 0.34; Side Effects, *t*(146)=1.38, *p*=0.171, 95% CI -0.30, 1.68. The confidence intervals for these mean differences are for scales with a maximum possible score of 5, 9 and 15, respectively; and therefore the 95% CIs span 19.8%, 11.8% and 13.2% of each scale-range, respectively. These spans therefore represent population effects for mean differences for which our sample had excellent statistical power (>.95) while for effects which might be considered small – differences in the region of 6% to 10% of the scale-range – our CIs imply modest statistical power ($\approx.$50).

Notice that in the second extract we have expressed the potential effect in as a proportion of the scale range. In doing so, we align with the reviewer’s comment that any designation of an effect as ‘small’, ‘medium’ or ‘large’ is either conventional or arbitrary. This description of the potential effect is much more conducive to reader’s exercising their own judgment in these matters. Personally, we would regard difference of 15-20% of these scales as important, and differences of around 6-10% as modest. However, these way of presenting the information allows readers to exercise their own judgment.

We respectfully disagree with the reviewer’s view that *p*-values around 0.15 might be taken as evidence of an effect. We prefer to adopt a more conservative and conventional interpretation of p-values, which is that we simply should not speculate about the possible presence or direction of an effect in such circumstances. This is very much in keeping with the weight of reviewer opinion in our experience, wherein we have regularly seen authors chastised by reviewers for describing effects with p = .07 or p = .08 as ‘approaching significance’ or ‘marginally significant’. We may indeed have missed a small effect – but p $\approx$ .15 cannot be taken as evidence that we should be confident the direction of any such effect, should it exist. This conservatism is in line with current concerns about reproducibility and replication in psychological science where many effects that have been identified with p = .03 or .04 have subsequently been found to be non-replicable (e.g., Munafo, Nosek, Bishop et al., 2017). Perhaps also relevant to mention here, the (non-significant) mean difference of around one-half is entirely consistent with the significant difference that we find and report whereby approximately an additional 50% of CE users use personal information as a source of information.

Related to these issues, the reviewer argues that we have not met one of the main objectives of our study: “One of the main objectives of this study was to clarify whether there were differences in the sources of knowledge on the safety of CE between its users and non-users.” This does not quite match with how we expressed our objective concerning sources of knowledge. We said that we aimed to compare sources of information between users and non-users, and demonstrably we have done this. Additionally, the reviewer seems to interpret our discussion to mean that the true difference in the number of information sources could have been one or two. However, we said that it is unlikely to be as large as one or two. We thought we had been clear in what we had written, however, hopefully the additional comment in the Results about this CI (see above) provides additional clarity on this.

Cumming, G. (2014). The new statistics: why and how. *Psychological Science*, *25*(1), 7–29. https://doi.org/10.1177/0956797613504966

Munafo, M. R., Nosek, B. A., Bishop, D. V. M., et al., (2017). A manifesto for reproducible science. *Nature Human Behaviour,* *1*:0021 DOI: 10.1038/s41562-016-0021
