# Supplementary material for: Understanding the relationship between safety beliefs and knowledge for cognitive enhancers in UK university students
Source: PLoS One. 2021 Jan 28;16(1):e0244865. doi: 10.1371/journal.pone.0244865 (PMC7842904; doi:10.1371/journal.pone.0244865)
Supplement: Attachment — Submitted filename: Response to Reviewers Dec 20.docx [file pone.0244865.s005.docx]

Thank you for the review of the previous version of our manuscript. We have now fully revised our manuscript in line with the reviewer comments. In tandem with these changes to the main manuscript, we now provide additional details of power calculations and statistical analyses in Supporting Information (S2). Please see below for our response to each reviewer point.

1. The analyzed sample consisted of 148 participants out of which 21% identified themselves as CE users. Although this data may be representative of the studied population, there is a disproportion in the size of the compared groups. This raises concerns about the homogeneity of variance of the compared groups that is an important assumption for the used ANOVAs. The manuscript should therefore explicitly mention how they dealt with this limitation and whether and why are the used statistical methods appropriate.

Thank you for this comment. Our Data Analysis section now confirm explicitly that equal variances were assumed for these analyses and point readers to our new Supporting Information (S2) that report a robustness checks of each between-subjects test of mean differences that do not assume equal variances:

For these and other between-subjects tests of mean difference we assume homogeneity of variance in the population. Analyses reported in the Supporting Information (S2) show that no conclusions change if this assumption is not made.

The Supporting Information (S2) explain that the assumption of equal variances in the population is reasonable because we had no *a priori* reason to depart from this assumption, and because the observed differences between groups in sample variance were small. Nonetheless, the point is well made that any issues arising from heterogeneity in variances are exacerbated when the sample sizes are unequal. Therefore, we think it prudent to include analyses in our Supporting Information (S2) which demonstrate that analyses that assume unequal variances yield the same conclusions as analysis that assume equal variances.

2. The different group sizes are also related to another, previously discussed, issue: the statistical power. When I used the widely recommended (e.g. Cumming, 2014) G*Power software (available at tiny.cc/gpower3) to calculate the power of the study given the provided data, the power was much lower than the one reported in the manuscript (0.95). Please note that I do not recommend post-hoc power calculation. However, it would considerably increase the quality of the manuscript, if the authors included information about the probability of finding predicted difference, given the study parameters, which is not be based only on the confidence intervals.

Thank you for suggesting that we use G*Power software. This has allowed us to provide details of statistical power in a format that will be more familiar to readers. We agree with the reviewer that there is limited value to a post hoc power calculation (e.g., based on the observed effect size). We have therefore based our power calculations on the power to detect effects in the population that we would not want to miss. These were specified in terms of unstandardised mean differences, because these have a meaningful and concrete interpretation in the context of our study (see Baguley, 2009). This required that we use sample data to estimate the standard deviation of scores in the population (as is almost always the case in the behavioural sciences). These calculations took account of the unequal sample sizes. Our Supporting Information (S2) describe this process in detail.

We have replaced the previous description of power in the Results with descriptions based the power calculations in G*Power. These confirm that most analyses had very good statistical power to detect the effects that we specified for the calculations.

On page 13, we now write:

Using G*Power software, we determined that our sample size was sufficient for 0.95 power to detect a mean difference of one additional data source in the population using a two-tailed test (see Supporting Information (S2) for full details). Therefore, if a difference of that scale or larger were to exist in the population, it is highly unlikely that we would fail to detect a difference.

And on page 15:

We used G*Power software to determine the sample sizes required for 0.8 power to detect an absolute mean difference of 10% of the scale-range for each knowledge scores. Our sample size exceeds the sample size required for this level of statistical power for the monitor and not safe scores, though an additional 56 participants would be required to achieve this high degree of power for the not safe scale (see Supporting Information (S2)).As indicated in these additions, fuller details are given in the Supporting Information (S2).

We have also added this short comment to the Limitations sub-section (page 21) because our re-calculation of power highlighted a potential additional route to improve measurement precision and statistical power.

Relatedly, we note here that the one analysis where we did not achieve 80% power to detect a large effect was the knowledge scale with the lowest degree of precision (5 questions, generating 6 levels of performance). Having fewer questions for this measure than the others may have reduced the signal-to-noise ratio associated with this measure, and reduced power accordingly. Therefore, future studies should – if feasible – aim for a more fine-grained measure of knowledge.

For transparency, we have appended the protocols for each power analysis at the end of this letter.

3. There are a few cases of misplaced punctuation and inconsistency in citation style.

We have checked the manuscript for punctuation and style. Additionally, we have corrected one rounding error in the reporting of an effect size (d = 0.40 corrected to d = 0.39 on page 13).

Kind regards,

Eleanor Dommett and colleagues

**Reference**

Baguley, T., 2009. Standardized or simple effect size: what should be reported? *British Journal of Psychology*, 100(3), 603-617.

**Protocols for power calculations using G*Power**

**1. Sample size required for 0.95 power to detect a mean difference of 1 additional information sources (estimated standardised effect of d = 0.834)**

*[1] -- Monday, December 14, 2020 -- 12:52:07*

**t tests** - Means: Difference between two independent means (two groups)

**Analysis:**	A priori: Compute required sample size

**Input:**	Tail(s)	=	Two

Effect size d	=	0.834

α err prob	=	0.05

Power (1-β err prob)	=	0.95

Allocation ratio N2/N1 =	3.9

**Output:**	Noncentrality parameter δ	= 3.6466543

Critical t	=	1.9806260

Df	=	116

Sample size group 1	=	24

Sample size group 2	=	94

Total sample size	=	118

Actual power	=	0.9511731

**2. Sample size required for 0.8 power to detect a mean difference in knowledge score (not safe) of 10% of the response scale (mean difference of 0.5, estimated standardised effect of d = 0.491).**

*[2] -- Monday, December 14, 2020 -- 13:03:29*

**t tests** - Means: Difference between two independent means (two groups)

**Analysis:**	A priori: Compute required sample size

**Input:**	Tail(s)	=	Two

Effect size d	=	0.491

α err prob	=	0.05

Power (1-β err prob)	=	0.80

Allocation ratio N2/N1	=	3.933

**Output:**	Noncentrality parameter δ	=	2.8102965

Critical t	=	1.9717774

Df	=	202

Sample size group 1	=	41

Sample size group 2	=	163

Total sample size	=	204

Actual power	=	0.7986900

**3. Sample size required for 0.8 power to detect a mean difference in knowledge score of 10% of the response scale (mean difference of 0.9, estimated standardised effect of d = 0.686).**

*[3] -- Monday, December 14, 2020 -- 13:05:00*

**t tests** - Means: Difference between two independent means (two groups)

**Analysis:**	A priori: Compute required sample size

**Input:**	Tail(s)	=	Two

Effect size d	=	0.686

α err prob	=	0.05

Power (1-β err prob)	=	0.8

Allocation ratio N2/N1	=	3.933

**Output:**	Noncentrality parameter δ	=	2.8150771

Critical t	=	1.9830375

Df	=	104

Sample size group 1	=	21

Sample size group 2	=	85

Total sample size	=	106

Actual power	=	0.7964569

**4. Sample size required for 0.8 power to detect a mean difference in knowledge score of 10% of the response scale (mean difference of 1.5, estimated standardised effect of d = 0.686).**

*[4] -- Monday, December 14, 2020 -- 13:06:57*

**t tests** - Means: Difference between two independent means (two groups)

**Analysis:**	A priori: Compute required sample size

**Input:**	Tail(s)	=	Two

Effect size d	=	0.612

α err prob	=	0.05

Power (1-β err prob)	=	0.8

Allocation ratio N2/N1	=	3.933

**Output:**	Noncentrality parameter δ	=	2.8362270

Critical t	=	1.9783804

Df	=	130

Sample size group 1	=	27

Sample size group 2	=	105

Total sample size	=	132

Actual power	=	0.8037949
